# 

**Published:** 1886-12

**Authors:** 


### CONTENTS, DEC. 1886, (VOL. II, NO. VI.)

ORIGINAL COMMUNICATIONS—Abortion, from the Standpoint of Personal Experience, by S. H. Stout, A. M., M. D., L. L. D., Cisco, Texas........................... 221

Hydrocephalus Complicating Breech Presentation, by E. J. Dœring, M. D., Chicago, Illinois............ 226

Veratrum Viride in Puerperal Eclampsia, by U. G. M. Walker, M. D., Tyler, Texas..................... 228

"Niguas," a Parasite of Northern Mexico, by C. K. Gregg, M. D., Ramos Arispe.................. .. 230

Dr. McLaughlin's Microbic Theory of Dengue, by R. Menger, M. D., San Antonio, Texas............ 232

SOCIETY NOTES.—Transactions Texas State Medical Association: Opinions of The Press.................... 237

Minutes of the Regular Annual Meeting of The West Texas Medical Association....................... 240

Organization of The "East Texas Medical Association," at Nacogdoches........................... 241

BIBLIOGRAPHY.—"That Prize Essay," by D. R. Wallace, M. D., Terrell, Texas............................242–258

MEDICAL NEWS AND MISCELLANY.—Personal—Dr. T. J. Tyner; An Important Step contemplated............ 259

Hypodermic Medication: Died. Mrs. Burger, Mrs. Stone.................................................... 260

"Practice." Found at last: The New Remedy for Tetanus.............................................. 261

Quarantine against Cholera: Married............... 261

